# The Effects of αvβ3 Integrin Blockage in Breast Tumor and Endothelial Cells under Hypoxia In Vitro

**DOI:** 10.3390/ijms23031745

**Published:** 2022-02-03

**Authors:** Bruna C. Casali, Larissa T. Gozzer, Matheus P. Baptista, Wanessa F. Altei, Heloisa S. Selistre-de-Araújo

**Affiliations:** 1 Departamento de Ciências Fisiológicas, Universidade Federal de São Carlos, São Carlos 13560-905, SP, Brazil; brunaccasali@yahoo.com.br (B.C.C.); larissagozzer@estudante.ufscar.br (L.T.G.); matheuspbaptista12@gmail.com (M.P.B.); 2 Programa de Pós-Graduação em Genética Evolutiva e Biologia Molecular, Universidade Federal de São Carlos, São Carlos 13560-905, SP, Brazil; 3 Departamento de Radioterapia, Hospital do Câncer de Barretos, Barretos 14784-400, SP, Brazil; wanessa.altei@hospitaldeamor.com.br; 4 Centro de Pesquisa em Oncologia Molecular, Hospital do Câncer de Barretos, Barretos 14784-400, SP, Brazil

**Keywords:** breast tumor, hypoxia, αvβ3 integrin blocking, cell migration, disintegrin, Dis*Ba*-01

## Abstract

Breast cancer is characterized by a hypoxic microenvironment inside the tumor mass, contributing to cell metastatic behavior. Hypoxia induces the expression of hypoxia-inducible factor (HIF-1α), a transcription factor for genes involved in angiogenesis and metastatic behavior, including the vascular endothelial growth factor (VEGF), matrix metalloproteinases (MMPs), and integrins. Integrin receptors play a key role in cell adhesion and migration, being considered targets for metastasis prevention. We investigated the migratory behavior of hypoxia-cultured triple-negative breast cancer cells (TNBC) and endothelial cells (HUVEC) upon αvβ3 integrin blocking with Dis*Ba*-01, an RGD disintegrin with high affinity to this integrin. Boyden chamber, HUVEC transmigration, and wound healing assays in the presence of Dis*Ba*-01 were performed in hypoxic conditions. Dis*Ba*-01 produced similar effects in the two oxygen conditions in the Boyden chamber and transmigration assays. In the wound healing assay, hypoxia abolished Dis*Ba*-01′s inhibitory effect on cell motility and decreased the MMP-9 activity of conditioned media. These results indicate that αvβ3 integrin function in cell motility depends on the assay and oxygen levels, and higher inhibitor concentrations may be necessary to achieve the same inhibitory effect as in normoxia. These versatile responses add more complexity to the role of the αvβ3 integrin during tumor progression.

## 1. Introduction

Despite the advances in diagnostics and treatment, breast cancer remains with high incidence and mortality, with 18.1 million new cases and 9.9 million deaths worldwide, being the main leading oncological cause of female deaths in 2020 [1]. Triple-negative breast cancer (TNBC) is characterized by the absence of estrogen receptors (ER), progesterone receptors (PR), and human epidermal growth factor type 2 receptor (HER2), resulting in a poor prognosis, since these cell types do not respond to conventional receptor-targeted therapies [2]. Moreover, TNBC cells are highly metastatic, using the tumor microenvironment and the extracellular matrix (ECM) as support for proliferation and spreading [3,4]. During tumor development, cancer cells induce collagen deposition in the surrounding microenvironment, increasing ECM stiffness, known as tumor fibrosis [5,6]. Solid tumors such as breast cancer are usually characterized by fibrosis and uncontrolled cell proliferation combined with abnormal vascularization, resulting in hypoxic areas in the middle of the tumor [7,8]. Patients with poorly oxygenated solid tumors have a higher risk of developing metastasis [9,10].

In hypoxic conditions, the hypoxia-induced factor (HIF-1) is the central molecule that triggers cellular responses. HIF-1 is composed of two subunits, HIF-1α and HIF-1β, whose interaction activates hypoxic response elements (HRE), promoting the expression of pro-angiogenic genes, primarily the vascular endothelial growth factor (VEGF) [11]. This response will stimulate tumor vascularization in order to provide better tumor nutrition; however, these new vessels are not well formed and present higher permeability compared to normal vessels. Defective tumor angiogenesis will also contribute to tumor cell transmigration across the endothelial barrier and to the spread of malignant cells through the body [9,10]. HIF-1α also induces matrix metalloproteinase (MMP) expression, such as gelatinases MMP-2 and MMP-9, which degrade the ECM, assisting in tumor cell migration [12,13]. Other HIF-1α targets of increased expression are some membrane receptors such as the integrins [14,15].

Integrins are transmembrane dimeric receptors formed by a noncovalent interaction between alpha and beta (αβ) subunits, being responsible for cell adhesion to the ECM [16]. Integrins are involved in a number of physiological processes, including chondrogenesis, axonal regeneration, and ECM remodeling [17,18,19]. Integrins have a critical role in cell migration, which is one of the main events in the metastatic cascade. To reach secondary sites, tumor cells must detach from the primary tumor, degrade and invade the ECM, and transmigrate through the endothelial barrier to finally intravasate to the blood or lymphatic vessels [20,21]. During these steps, integrins mediate adhesion foci assembly and disassembly, supporting cell movement and providing directionality [22]. Furthermore, the recycling of integrins is required for successful cell migration [21]. Integrin activation upon ECM binding triggers intracellular signaling cascades of several kinases, including focal adhesion kinase (FAK), mitogen-activated kinase (MAPK), and extracellular signal-regulated kinase (ERK) [23]. In endothelial cells, integrin activation is linked to the activation of the VEGF/VEGFR2 axis; therefore, integrins are intimately related to the control of angiogenesis [24,25]. This integrin–ECM crosstalk is a central player in the migratory ability of cells; therefore, integrins have become interesting targets for metastasis prevention or treatment [26,27].

Integrin expression changes according to the type of tumor and the disease stage [28]. Integrins α5β1, α2β1, α6β1, αvβ5, α5β3, and, in particular, αvβ3 have essential roles in tumorigenesis and angiogenesis [29]. The αvβ3 integrin recognizes RGD ligands present in the ECM proteins such as fibronectin and vitronectin, promoting cell motility and metastasis [30]. Recently, the αvβ3 integrin was demonstrated to be translationally activated in hypoxia, resulting in activation of the epithelial–mesenchymal transition program and cell migration, and increased metastatic behavior [31]. An integrin inhibitor, cilengitide, inhibited the αvβ3 and αvβ5 integrins and tumor progression in a number of pre-clinical assays that stimulated its testing in clinical trials [32]. Cilengitide, however, has not increased glioblastoma patient survival [33,34] or decreased the number of metastases [35]. Cilengitide’s failure may be related to the dose, tumor type, or the lack of deeper knowledge on the integrins’ molecular mechanisms of action [36]. Therefore, a better understanding of integrin function and searches for new integrin antagonists are of evident interest [37].

Disintegrins are natural integrin inhibitors used as tools in the design of new anti-cancer therapies [38]. Disintegrins such as bothrasperin from *Bothrops asper* and veridistatin from *Crotalus viridis* inhibit the adhesion of melanoma cells and migration of murine breast cancer cells, respectively [39]. Most disintegrins exhibit an adhesive motif, such as RGD, ECD, or KTS, that binds to specific integrins. Accurhagin-c, an ECD disintegrin from *Agkistrodon acutus*, is a αV/α5 antagonist that prevents the migration and invasion of endothelial cells and decreases B16F10 proliferation [40].

Dis*Ba*-01 is an RGD recombinant disintegrin from *Bothrops alternatus* with high affinity to the αvβ3 integrin (K*_D_* = 1.6 **×** 10^−7^ M), with in vivo anti-angiogenic, anti-metastatic, and anti-thrombotic properties [41]. Dis*Ba*-01 is around 100-times more specific for the αvβ3 integrin than α5β1 (K*_D_* = 7.62 × 10^−5^ M) [42]. Dis*Ba*-01 inhibits cell proliferation and migration in a number of cell lines in vitro [41,42,43,44], and there is crosstalk between the αvβ3 integrin and the vascular endothelial growth factor receptor-2 (VEGFR2) in HUVECs [45,46]. Dis*Ba*-01 impaired the directionality of oral squamous cell carcinoma migration [42]. All in vitro studies with Dis*Ba*-01 were performed in normoxia, a very different condition from the one found inside solid tumors.

Here, we investigate the migration of breast cancer MDA-MB-231 cells and endothelial cells in hypoxia using some in vitro models, focusing on the effect of αvβ3 integrin blocking upon treatment with Dis*Ba*-01. Due to the essential role of the αvβ3 integrin in metastatic spreading, our results indicate the distinct behavior of the tumor and endothelial cells upon αvβ3 integrin blockade, depending on the migration assay and oxygen condition. These results might be of relevance when considering testing integrin inhibitors in clinical trials for solid tumors.

## 2. Results

### 2.1. Blocking αvβ3 Integrin Inhibits MDA-MB-231 Cell Migration in Normoxia and Hypoxia

**Figure 1:**
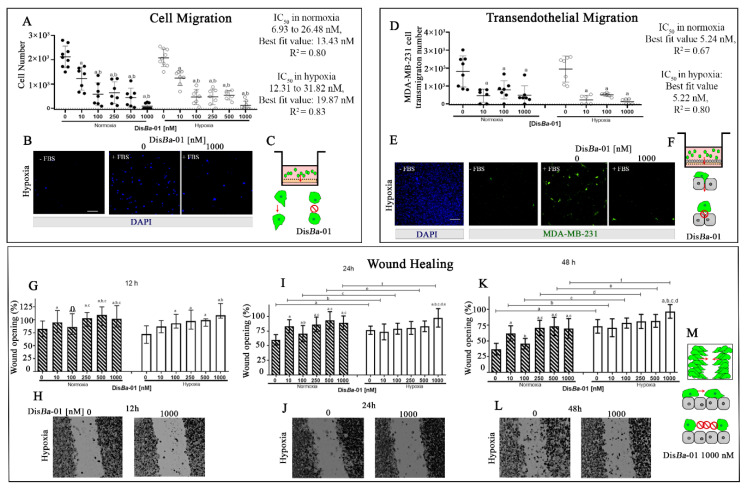
Inhibition of MDA-MB-231 cell migration by αvβ3 integrin blocking in normoxia and hypoxia. (**A**–**C**) Boyden chamber assay, MDA-MB-231 cells treated with indicated Dis*Ba*-01 concentrations. (**A**) Migrated cells in absence or presence of Dis*Ba*-01 in normoxia and hypoxia. Values were compared to negative control (without chemoattractant). (**B**) Representative images of migrating cells treated or not with Dis*Ba*-01 in hypoxia. (**D**–**F**) Transendothelial migration of CFSE-labeled MDA-MB-231 cells in a HUVEC layer. (**D**) Transmigrated cells in absence and presence of Dis*Ba*-01 in normoxia and hypoxia. Values were compared to negative control (without chemoattractant). (**A**,**D**) graphics represent mean ± SD. (**E**) Representative images of transmigrating cells treated or not with Dis*Ba*-01 in hypoxia. (**C**,**F**) Graphical summary of the two assays. (**G**–**M**) Wound healing assay of MDA-MB-231 cells in the presence of Dis*Ba*-01 in normoxia and hypoxia for 12 (**G**–**H**), 24 (**I**–**J**), and 48 (**K**–**L**) hours. (**M**) Graphical summary of the wound healing assay in the presence of Dis*Ba*-01. (**G**,**I**,**K**) graphics represent median ± SD. Letters over bars mean: *a*, significantly different from control; *b*, significantly different from 10 nM; *c*, from 100 nM; *d*, from 250 nM, and *e*, from 500 nM (mean ± SD). All experiments were performed in triplicate from three independent assays (*n* = 3, *p* < 0.05). Scale bar: 100 μm. Red arrows in graphical summary represent the direction of migration.

To study the role of the αvβ3 integrin in cell motility under hypoxia, we used three different migration models (transwell, endothelial transmigration, and wound healing) in hypoxia and in the presence or not of a specific antagonist, Dis*Ba*-01. The same assays were performed in parallel under normoxic conditions for comparison. MDA-MB-231 cell migration in the Boyden chamber was inhibited by Dis*Ba*-01 in a concentration-dependent way and in a similar way in the two oxygen conditions (Figure 1A–C). The IC50 values were 13.43 nM and 19.87 nM (*p* = 0.97) in normoxia and in hypoxia, respectively, indicating a small difference between the two conditions. Representative images of the analyzed membranes are depicted in Figure 1B.

We further addressed the role of the αvβ3 integrin in a transendothelial migration assay. MDA-MB-231 cells in suspension were treated with Dis*Ba*-01 and placed inside the insert to transmigrate through a HUVEC monolayer (Figure 1D–F). Dis*Ba*-01 inhibited transmigration in normoxia and hypoxia, with IC50 values of 5.24 nM and 5.22 nM, respectively, revealing no significant differences between the two oxygen conditions. Interestingly, 10 nM Dis*Ba*-01 induced maximal inhibition in the two oxygen conditions in the transmigration assay, different from the effect observed in the Boyden chamber migration assay, where the same inhibitory effect was observed only for the 1000 nM Dis*Ba*-01 concentration. Representative images of CFSE-labeled MDA-MB-231 cells after transmigration are shown in Figure 1E.

The wound healing assay was performed at three time points. After 12 h, there was no difference between normoxia and hypoxia (Figure 1G–H); however, after 24 and 48 h, there were significant differences between the two conditions (Figure 1I–L). Hypoxia impaired wound closure in Dis*Ba*-01-treated and non-treated cells. Moreover, Dis*Ba*-01′s inhibitory effect was detected in normoxia for all tested concentrations. Conversely, Dis*Ba*-01 was effective only at its highest concentration (1000 nM) in hypoxia (Figure 1I–K) after 24 and 48 h. Collectively, these results indicate that the αvβ3 integrin has a critical role for MDA-MD-231 cell migration since its inhibition significantly impairs chemotaxis. On the other hand, in the case of the wound healing assay, motility without a chemoattractant is strongly affected by lower oxygen conditions.

### 2.2. MMP Levels in the Conditioned Media from Cell Migration Assays

**Figure 2:**
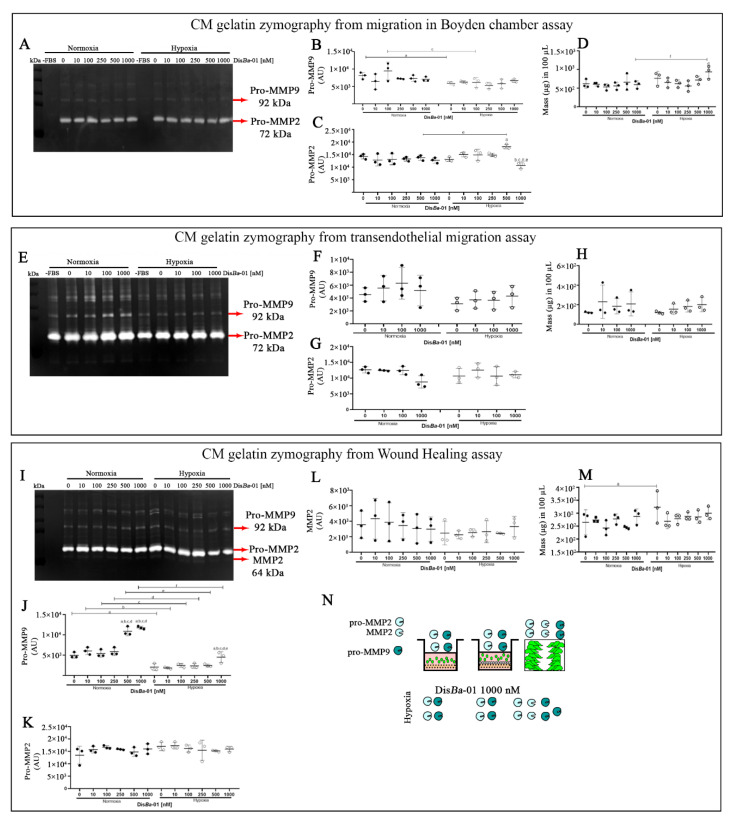
MMP-2 and MMP-9 levels in the conditioned media (CM) from MDA-MB-231 cell migration assays. (**A**,**E**,**I**) Representative zymographs of CM from transwell, transmigration, and wound healing assays; (**B**,**F**,**J**) Quantification of pro-MMP-9 levels by densitometry; (**C**,**G**,**K**,**L**) Quantification of pro-MMP-2 and active MMP-2 levels by densitometry; (**D**,**H**,**M**), CM total protein concentration. (**N**) Graphical summary of the assays. Experiments were performed in triplicate with three independent assays (*n* = 3). The results (mean ± SD) were compared using two-way ANOVA followed by Tukey’s test (*p* < 0.05). Graphic letters *a*, *b*, *c*, *d*, and *e* represent comparisons among 0, 10,100, 250, 500, and 1000 nM of Dis*Ba*-01, respectively.

We next tested MMP activity in the conditioned media (CM) from the independent assays by gelatin zymography (Figure 2). Our hypothesis was based on our previous studies in normoxia, where we demonstrated that Dis*Ba*-01 decreased MMP-2 activity, which would contribute to the inhibition of cell migration [42,43]. The pattern of MMP activity in the CM from the transwell assay was similar in normoxia and hypoxia (Figure 2A), with slight differences between the two conditions. The main bands detected and quantified were pro-MMP-9 and pro-MMP-2. The levels of pro-MMP-9 were higher in the control samples in normoxia compared to hypoxia (Figure 2B). A tendency for pro-MMP-9 to decrease upon Dis*Ba*-01 treatment was observed only for the 100 nM concentration in hypoxia (Figure 2B). Dis*Ba*-01′s effect was more pronounced on the pro-MMP-2 levels, mostly in hypoxia and only for the highest concentrations (Figure 2C).

We did not observe any significant differences in MMP pattern in the CM from the transmigration assays in normoxia or hypoxia. Dis*Ba*-01 did not affect MMP activity in either condition (Figure 2E–H). Conversely, hypoxia decreased the levels of pro-MMP-9 in the CM from the wound healing assay (Figure 2I,J), without changes in pro-MMP-2 bands (Figure 2K,L). Only the highest Dis*Ba*-01 concentrations (500 and 1000 nM) increased the pro-MMP-9 levels in both normoxia and hypoxia conditions (Figure 2J). There were no differences in CM total protein concentration from all the assays in the two oxygen conditions (Figure 2D,H,M). We conclude that hypoxia negatively affected MMP-9 expression in the wound healing assay, independently of αvβ3 integrin inhibition.

### 2.3. DisBa-01′s Effects on HUVEC Tube Formation Ability in Normoxia and Hypoxia

**Figure 3:**
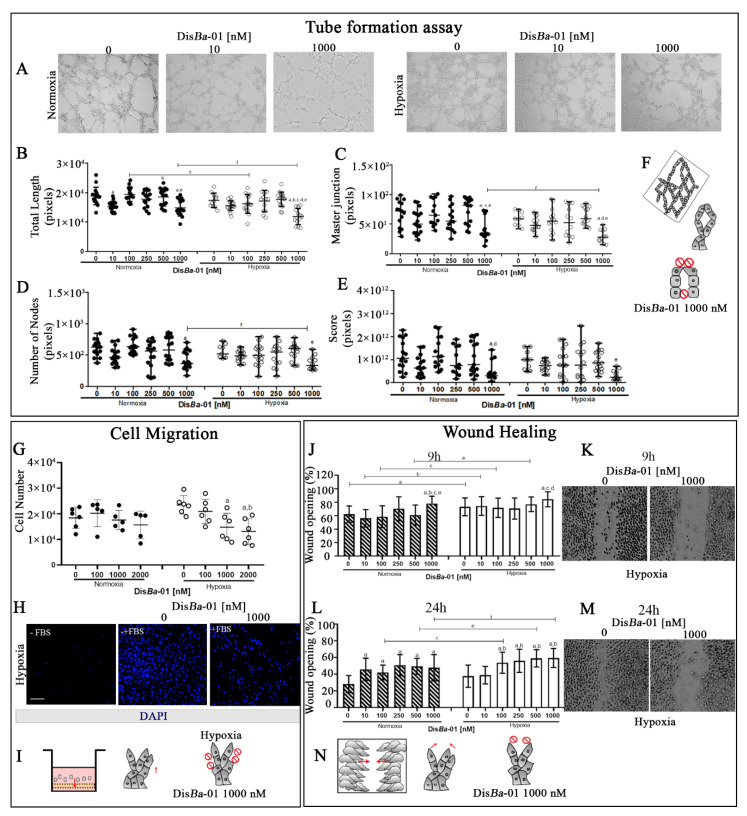
Inhibition of tube formation and cell migration of Dis*Ba*-01-treated HUVECs in normoxia and hypoxia. (**A**–**F**) Tube formation assay. Representative images of HUVECs in the indicated conditions (**A**), total length (**B**), master junctions (**C**), number of nodes (**D**), and score (**E**) by pixel quantification of Dis*Ba*-01-treated HUVECs in the two oxygen conditions. Experiments were performed in triplicate of three independent assays (*n* = 3, *p* < 0.05). (**F**) Graphical summary of the assay. (**G**–**H**), Boyden chamber migration assay of Dis*Ba*-01-treated HUVECs. Values were compared to negative control (without chemoattractant) (**G**). Representative images of migrating cells treated or not with Dis*Ba*-01 in hypoxia (**H**). Scale bar: 100 µm. (**J**–**N**) HUVEC wound healing assay. Percentage (mean ± SD) of wound opening in indicated concentrations of Dis*Ba*-01 in normoxia and hypoxia after 9 and 24 h (**J**,**L**). Representative images of scratches in hypoxia (**K**,**M**). (**I**,**N**) Graphical summary of the two migration assays. Graphic letters *a*, *b*, *c*, *d*, *e*, and *f* represent comparisons between 0, 10,100, 250, 500, and 1000 nM of Dis*Ba*-01, respectively. All experiments were performed in triplicate from three independent assays (*n* = 3, *p* < 0.05). Red arrows in graphical summary represent the direction of migration.

One of the initial steps of tumor angiogenesis is tube development. To address the effect of hypoxia in this process, HUVECs were grown on GFR Matrigel for the development of a capillary-like network. Parameters such as total length, master junctions, number of nodes, and score (area × total branching × number of meshes) were measured in normoxia and hypoxia in the presence or absence of Dis*Ba*-01. The total length of tubes, the number of nodes, and master junctions were reduced by αvβ3 integrin blocking by Dis*Ba*-01 only at its highest concentration (1000 nM), both in normoxia and hypoxia (Figure 3A–E). Representative images of this assay are shown in Figure 3A. We conclude that hypoxia does not significantly inhibit tube formation and higher concentrations of integrin inhibitors are necessary to inhibit this process.

We also tested the ability of Dis*Ba*-01 to inhibit HUVEC migration in the Boyden chamber and wound healing assays. Dis*Ba*-01 had no effect in normoxia, and it inhibited HUVEC migration in the Boyden chamber assay only at high concentrations in hypoxia (Figure 3G,H). Results of the wound healing assay were distinct after 9 and 24 h. After 9 h, Dis*Ba*-01 was effective in inhibiting the closure only at its highest concentration, both in normoxia and hypoxia (Figure 3J,K). After 24 h, however, all Dis*Ba*-01 concentrations inhibited wound healing in the two conditions, with the exception of the 10 nM concentration in hypoxia (Figure 3L,M). We conclude that hypoxia inhibits wound closure and high concentrations of Dis*Ba*-01 are needed for integrin inhibition in this condition.

### 2.4. Levels of β3 Integrin Subunit Change Depending on the Cell Type and Oxygenation

**Figure 4:**
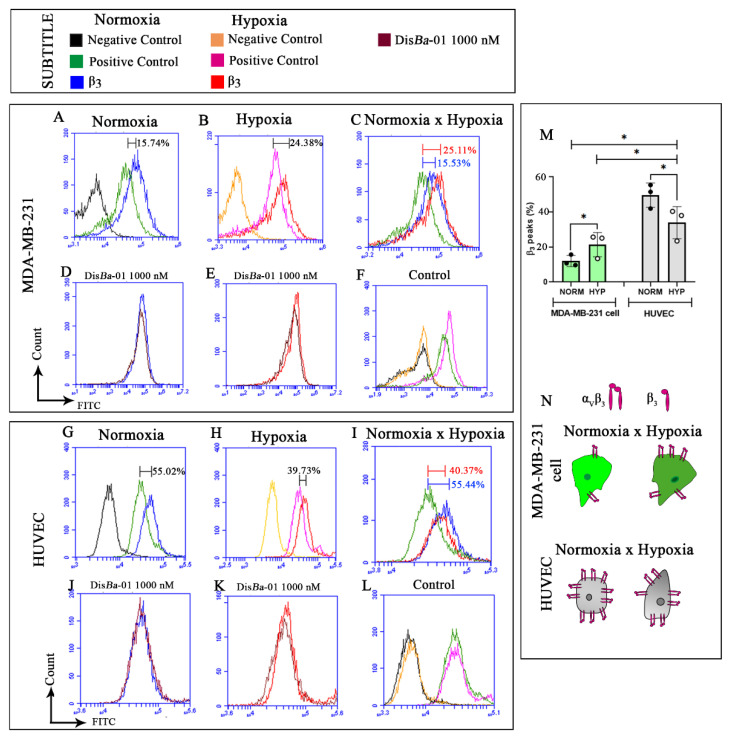
Profile of β3 integrin subunit in MDA-MB-231 and HUVECs in normoxia and hypoxia. (**A**–**F**) Detection of β3 integrin subunit in MDA-MB-231 cells in normoxia (**A**), in hypoxia (**B**), and merge of (**A**) and (**B**) (**C**). Detection of β3 integrin in MDA-MB-231 cells after Dis*Ba*-01 treatment in normoxia (**D**), and in hypoxia (**E**), and negative and positive controls in normoxia and hypoxia (**F**). (**G**–**L**) Detection of β3 integrin subunit in HUVECs in normoxia (**G**), in hypoxia (**H**), and merge of (**G**) and (**H**); (**I**) HUVEC β3 integrin content after Dis*Ba*-01 treatment in normoxia (**J**) and in hypoxia (**K**), and negative and positive controls in normoxia and hypoxia (**L**). (**M**) Data quantification and statistics for MDA-MB-231 cells and HUVECs under normoxia and hypoxia. * means statistical differences between MDA-MB-231 cells (green bars) and HUVEC (gray bars) in normoxia and hypoxia. (**N**) Graphical summary of β3 integrin subunit profile in MDA-MB-231 cells and HUVECs with or without Dis*Ba*-01 in normoxia and hypoxia. Experiments were performed in triplicate from three independent assays (*n* = 3, *p* < 0.05).

Since cells can change their integrin content according to the signals from the milieu, we next analyzed whether hypoxia could affect the expression of the β3 integrin subunit by flow cytometry. MDA-MB-231 cells presented around 15% of β3 integrin subunit in normoxia, but this value increased by almost 10% (24%) in hypoxia (Figure 4A–C). Dis*Ba*-01 treatment had no effect in both oxygen conditions (Figure 4D,E). Controls were similar in normoxia and hypoxia (Figure 4F).

Results in HUVECs showed the opposite. The expression of the β3 subunit integrin in HUVECs was approximately 55% in normoxia and 40% hypoxia, a decrease of approximately 15% in the lower oxygen condition (Figure 4G–I). Similarly to MDA-MB-231 cells, Dis*Ba*-01 treatment did not alter β3 integrin content in HUVECs in normoxia or in hypoxia (Figure 4J,K). Controls were similar in normoxia and hypoxia (Figure 4L).

### 2.5. Blockage of αvβ3 Integrin by DisBa-01 Disturbs MDA-MB-231 Cells and HUVEC Morphology in Normoxia and Hypoxia without Inducing Apoptosis

**Figure 5:**
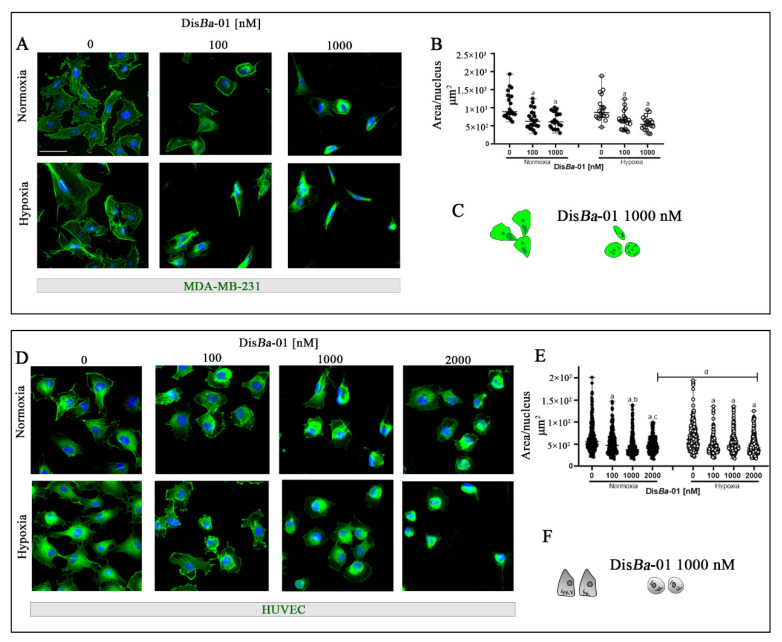
The morphology of MDA-MB-231 cells and HUVEC changes upon αvβ3 integrin blocking by Dis*Ba*-01 in normoxia and hypoxia. (**A**) MDA-MB-231 cells treated with Dis*Ba*-01 for 4 h. (**B**) Graphic represents the sum of cell total area (µm2) divided by the number of nuclei in the two conditions. Analysis was performed using ImageJ after cell staining in 100 cells per well. (**D**) HUVECs were treated with Dis*Ba*-01 for 4 h. (**E**) Graphic represents cell area (µm2) divided by the number of nuclei in normoxia and hypoxia. Experiments were performed in duplicate or triplicate with three independent assays (*n* = 3). Results for MDA-MB-231 cells were compared using two-way ANOVA followed by Tukey’s test (*p* < 0.05). HUVEC results were compared using the Kruskal–Wallis one-way analysis of variance on ranks post hoc Dunn test and all data (*p* < 0.05). Results are shown as median with range of variation. Graphic letters *a*, *b*, *c*, and *d* represent comparisons among 0, 100, 1000, and 2000 nM of Dis*Ba*-01, respectively. Scale bar = 50 μm. Graphical summary of MDA-MB-231 (**C**) and HUVEC (**F**) morphology with and without Dis*Ba*-01 in normoxia and hypoxia.

Cell migration can be impaired due to loose cell adhesions by the disassembly of the actin cytoskeleton and interruption of binding between extracellular matrix proteins and integrins. We therefore investigated possible changes in the morphology of MDA-MB-231 cells and HUVECs after Dis*Ba*-01 treatment in hypoxia compared with normoxia. As expected, Dis*Ba*-01 decreased the cell total area/nucleus ratio at the tested concentrations similarly at the two oxygen conditions for the MDA-MB-231 cells (Figure 5A,B). Similar results were found for HUVECs with only a minor difference observed. The highest Dis*Ba*-01 concentration (2 μM) was more effective in normoxia than hypoxia (Figure 5D,E).

The possibility of either hypoxia or Dis*Ba*-01 treatment to induce apoptosis was investigated by flow cytometry. Since wound healing assays were performed in the presence of mitomycin-c to avoid measuring cell proliferation instead of migration, we tested cells for apoptosis in the presence or not of mitomycin-c. Dis*Ba*-01 did not induce apoptosis, as demonstrated by the PE-annexin V assays, either in normoxia or in hypoxia; however, hypoxia induced apoptosis in approximately 10% of cells, but only in the presence of mitomycin-c (Supplementary Figures S1–S4). Therefore, we conclude that the inhibition of the αvβ3 integrin by Dis*Ba*-01 does not induce apoptosis in hypoxia or normoxia. Despite the loose adhesions, cells remain attached and do not die. 

## 3. Discussion

Cell migration is critical for tumor angiogenesis and metastasis, and the αvβ3 integrin plays a critical role in these two processes. Antagonists of the αvβ3 integrin strongly inhibit cell migration and cell directionality as well [22,42]. However, it is not well understood why the good results obtained in pre-clinical assays are not reproduced in vivo when translated into clinical trials [36]. One of the reasons for the low effectiveness of such inhibitors could be the lack of deeper knowledge about the microenvironment within a solid tumor, often under hypoxic conditions. In the present paper, we have studied the role of the αvβ3 integrin in a hypoxic milieu using a strong inhibitor of this receptor in a set of migration assays. We have previously determined that Dis*Ba*-01 has approximately 100-times more affinity to the αvβ3 than α5β1 integrin, another RGD-binding receptor involved in cell migration [42]. This specificity allowed us to conclude that the observed cellular effects upon Dis*Ba*-01 treatment are mostly due to the αvβ3 integrin, at least at the lowest concentrations.

Dis*Ba*-01 was previously demonstrated to inhibit HUVEC and 4T1BM cell migration in normoxia [45,47] but it was never tested in hypoxia as we show here. Intriguingly, inhibition results varied depending on the assay. In the Boyden chamber assay, Dis*Ba*-01 inhibited the motility of MDA-MB-231 cells regardless of the oxygen level. The same effect was observed in the endothelial transmigration assay; however, in hypoxia, the maximum inhibitory effect was achieved with the lowest Dis*Ba*-01 concentration. This result may be a consequence of the increased levels of tumor cell β3 integrin in hypoxia and suggests a key role for endothelial αvβ3 integrin in the interaction with tumor cells during extravasation. Despite the high Dis*Ba*-01 specificity to the αvβ3 integrin, other surface proteins may be overexpressed in HUVECs under hypoxia and could additionally interfere in tumor cell extravasation. More studies are needed to confirm this possibility.

The most significant effect of hypoxia was observed in the wound healing assay after 24 and 48 h of incubation, where only the highest Dis*Ba*-01 concentration was effective. One of the main differences between the wound healing and the transwell assays relies on the lack of a chemoattractant that provides directionality for the migrating cell. Since the αvβ3 integrin is critically involved in movement direction [42], this assay proved to be more sensitive to Dis*Ba*-01, highlighting the effect on hypoxia.

A previous work demonstrated that the β3 integrin is translationally activated under hypoxia [31]. In this paper, the authors explored both the transcriptome and the translatome of MDA-MB-231 cells in hypoxia compared to normoxia and identified the β3 integrin as a critical target. Moreover, silencing of ITGB3 gene expression inhibited cell migration in a wound healing assay in hypoxia but not in normoxia [31]. Collectively, these results and ours suggest that hypoxia activates the β3 integrin and therefore higher concentrations of the inhibitor may be necessary to produce an effective inhibitory response.

Breast tumor cells release MMPs to the extracellular matrix. These proteolytic enzymes, including the gelatinases, have a key role in degrading ECM proteins, assisting in migration and invasion in the tumor microenvironment [48,49]. Furthermore, integrins are directly associated with MMP control [50]. Tumor cells usually express high levels of MMP-9, which supports cell motility during invasion [51]. The αvβ3 integrin activates MMP-2- and MMP-9-dependent pathways in breast cancer metastasis [52]. MMP-2 is a target for HIF-1α that intermediates endothelial migration and angiogenesis in hypoxia [53]. On the other hand, decreased MMP-9 levels in breast tumors are associated with tissue fibrosis, a common finding in this disease [54]. In this work, we demonstrated the distinct profiles of MMP-2 and MMP-9 from TNBC migration assays. Our results show that a hypoxic environment impairs MMP-9 upregulation in tumor cells. Decreased MMP-9 activity was previously correlated with hypoxia and matrix stiffness in breast cancer patients [54]. Expression of constitutively activated αvβ3 integrin in metastatic variants of TNBC MDA-MB-435 strongly increased migration due to elevated levels of MMP-9 [55]. Furthermore, the role of some members of the ADAM (A Disintegrin And Metalloproteases) protein family in TNBC cell migration has been previously demonstrated. For instance, ADAM8 has a key role in TNBC transendothelial migration by promoting the upregulation of MMP-9 [49]. These results are in agreement with ours and confirm the controlling role of integrins on MMPs.

Previous studies have shown the inhibitory effects on cell migration of other snake venom-derived proteins, including RGD disintegrins such as r-majostin from *Crotalus scutulaus scutulatus* and r-virisdistatin from *Crotalus viridis viridis* [56], tzabcanin from *C.simus* [57], dabmaurin–1 from *Daboia mauritanica* [58], and disintegrins from *Crotalus totonacus* [59] and *Bothrops alternatus* [60] for different types of tumor cells. These studies, however, were performed in normoxia only. Non-RGD disintegrins from *Crotalus durissus colineatus* inhibited MDA-MB-231 migration in a wound healing assay after 24 h in normoxia [61]. 

Angiogenesis is the process of producing new vessels to supply oxygen and nutrients to meet increasing tissue demands, such as that which occurs in solid tumors. Angiogenesis can be mimicked by the tube formation assay on Matrigel, where endothelial cells form tube-like structures. The composition and variability of the Matrigel affect cell growth and differentiation [62,63]. We have previously demonstrated that Dis*Ba*-01 inhibits tube formation in Matrigel in normoxia, even in the presence of exogenous VEGF [45]. Here, we show that Dis*Ba*-01′s effects are attenuated in the tube formation assay under hypoxia. Only the highest Dis*Ba*-01 concentration inhibits tube formation in hypoxia in GFR Matrigel. These results indicate that high concentrations of integrin inhibitors are required to halt angiogenesis in solid tumors.

Dis*Ba*-01 treatment strongly affects cell morphology, with decreased stress fibers, suggesting a possible loss of adherence upon αvβ3 integrin inhibition. In this case, cells would go into apoptosis; however, cytometry analysis showed that Dis*Ba*-01 does not induce apoptosis. We have previously reported that Dis*Ba*-01 activates the autophagy program instead of apoptosis, at least during the first 24 h, and cells remain attached, probably by using other adhesion receptors [47]. This study was carried out with 4T1BM cells, a murine TNBC cell line highly metastatic to the brain, but we believe that the same may happen with the cells used in the present work. The key role of the αvβ3 integrin in cell migration is not to support strong adhesions but to provide directionality for a moving cell, as previously reported by us and others [22,42]. Dis*Ba*-01′s effects on MDA-MB-231 cells and HUVEC morphology are independent of the oxygenation condition.

In conclusion, our results indicate that inhibiting the αvβ3 integrin in hypoxic conditions may demand higher inhibitor concentrations. Our data may be useful considering other types of cancer besides breast tumors, because integrins have been described as having a key role in different tumor types, including colorectal carcinoma [64]. Of course, we have to consider that each cell type may respond differently to hypoxia or to integrin inhibitors. The results described here can be helpful in the design of new pre-clinical and clinical studies targeting the integrins.

## 4. Materials and Methods

### 4.1. DisBa-01 Expression and Purification

The expression and purification of Dis*Ba*-01 was performed as described by [41]. Briefly, *E. coli* BL21(DE3) was transformed with plasmid pet28(a)Dis*Ba*-01. Protein expression was induced for 3 h, followed by lysis and purification in three steps: affinity chromatography (HIS-Select^®^ HF Nickel Affinity Gel, Sigma-Aldrich, Code: P6611), size-exclusion chromatography (Superdex 75 10/300 GL, GE Healthcare, Code: 17-5174-01, Uppsala, Sweden), and anion exchange chromatography (Mono-Q 5/50 GL, GE Healthcare, Code: 17-516601, Uppsala, Sweden). Total protein was determined by colorimetric detection of bicinchoninic acid assay (Pierce BCA Protein Assay, Thermo Scientific, Catalog Number: 23225, U.S.).

### 4.2. Cells and Cell Culture

Triple-negative breast tumor cells (MDA-MB-231) and human umbilical vein endothelial cells (HUVECs, 8 to 20 passages) were from ATCC. Both cell lines were maintained in Dulbecco’s modified Eagle’s medium (DMEM, Vitrocell, Vitrocell, Campinas, SP, Brazil) supplemented with 10% (*v*/*v*) fetal bovine serum (FBS, Vitrocell, Campinas, SP, Brazil), penicillin (100 IU/mL), streptomycin (100 mg/mL), and L-glutamine (2 mM), in a humidified environment with 5% CO_2_ at 37 °C. Subcultures were performed using trypsin and trypan blue stain solution (0.4%, Sigma Aldrich, St. Louis, MO, USA) on a TC20 automated cell counter (Bio-Rad, Hercules, CA, USA). Cells in experiments were maintained in 20% O_2_ and 5% CO_2_ (normoxia) and an incubator chamber (H35 Hypoxystation, Don Whitley Sci., Bingley, UK) with a gas mixture containing 1% O_2_ and 5% CO_2_ (hypoxia), both at 37 °C.

### 4.3. Transwell Boyden Chamber Assay

Chemotaxis assays were performed to assess MDA-MB-231 cell migration upon αvβ3 integrin blocking by Dis*Ba*-01. For transwell assays, a 24-well insert, ThinCert™ translucent PET membrane, 8.0 μm pore (Greiner Bio-one^®^, Frickenhausen, Germany) were used. MDA-MB-231 cells (1 × 10 ^5^) in medium without serum were treated with Dis*Ba*-01 for 30 min at room temperature and inserted into the upper part of the Boyden chamber. The lower chamber contained medium plus 10% SFB. The system was incubated for 16 h (MDA-MB-231 cells) or 24 h (HUVEC) at 37 °C in normoxic and hypoxic conditions. Filters were fixed with 3.7% paraformaldehyde and the remaining cells on the upper surface were removed using a cotton swab. The nuclei of migrating cells were stained with 0.7 ng/µL DAPI solution (Thermo Fisher Scientific, Waltham, MA, USA, Catalog Number: 62248). Membranes were assembled on a microscope slide for automated cell counting in an ImageXpress Micro microscope (Molecular Devices, San Jose, CA, USA) under 10× magnification with the Meta-X-press software, and quantified using the Multi Wavelength Cell Scoring.

### 4.4. Transendothelial Cell Migration Assay

To evaluate MDA-MB-231 cell migration through a layer of endothelial cells, 8 × 10^4^ HUVECs were subcultured onto 8.0 μm pore 12-well inserts (Greiner Bio-one^®^, Frickenhausen, Germany Catalog number: C34554) with serum in the upper and lower chambers for 24 h in 5% CO_2_ at 37 °C. Then, MDA-MB-231 cells (0.6 × 10^5^) were labeled with Cell Trace TM CFSE (Thermo Fisher Scientific, Waltham, MA, USA, Catalog number: C34554). MDA-MB-231 cells were treated with Dis*Ba*-01 in serum-free medium for 30 min at room temperature, and then allowed to transmigrate through the endothelial layer for 16 h at 37 °C in a normoxic and hypoxic environment. The lower chamber contained medium plus 10% SFB. Filters were fixed with 3.7% paraformaldehyde and the remaining cells on the upper surface were removed using a cotton swab. The nuclei of migrated cells were stained with 0.7 ng/µL DAPI solution (Thermo Fisher Scientific, Waltham, MA, USA, Catalog Number: 62248). Membranes were assembled on a microscope slide for automated cell counting in an ImageXpress Micro microscope (Molecular Devices San Jose, CA, USA) under 10× magnification with the Meta-X-press software, quantified using the Multi Wavelength Cell Scoring.

### 4.5. Wound Healing Assay

MDA-MB-231 cells (1 × 10^5^) and HUVEC (1 × 10^5^) were seeded in a 24-well culture plate for 48 and 24 h, respectively. The confluent monolayer was wounded using a sterile 200 µL pipette tip to generate a cell-free area. Then, cells were treated with 10 µg/mL mitomycin-c (Sigma, St. Louis, MO, USA, Code:M4287) for 4 h, followed by washing 2× with PBS. Cells were treated with Dis*Ba*-01 in medium containing 10% FBS and incubated in normoxia and hypoxia for 24 h. The images were captured using an inverted microscope (Axio Vert.A1 Zeiss—AxioCam MRc Zeiss camera, Oberkochen, Germany) using the AxionVision Rel.4.8 software of a Vert.A1 microscope (Zeiss) in a 10× magnifying glass in three areas each well. Cell migration was analyzed through ImageJ v.1.52a [65] software considering the percentage of wound opening border: = Δh × 100/T0, where Δh is the area of the wound measured at different times and T0 is the average of the area of the wound measured immediately after scratching.

### 4.6. Zymography Assay

The conditioned media from the transwell Boyden chamber, transendothelial, and wound healing assays with MDA-MB-231 cells were analyzed for their MMP content by gelatin zymography. Culture medium was collected, protein quantified, and incubated in sample buffer under non-reducing conditions. Samples were resolved on a 10% polyacrylamide gel containing 0.1% gelatin at 4 °C. Gels were washed two times with 2.5% Triton Χ-100 and incubated at 37 °C for 18 h in 50 mM Tris buffer, pH 8.0, 5 mM CaCl_2_, 0.02% NaN_3_, and 10 mM ZnCl_2_. After staining with Coomassie Blue R-250 and destaining with acetic acid:methanol:water (1:4:5), the clear bands were quantified by densitometry using ImageJ software. MMP-2 and MMP-9 were represented in arbitrary units (AU).

### 4.7. Tube Formation Assay

The tube formation assay on Matrigel (Growth Factor Reduced—GFR, Product Number: 354230, Corning, NY, USA) was performed to evaluate the ability of Dis*Ba*-01 in inhibiting angiogenesis after 10 h incubation under hypoxic conditions. Firstly, HUVECs (3 × 10^4^ cells) were treated for 30 min with Dis*Ba*-01 and plated on 1:1 Matrigel dilution (35 µL/well) in 0.5% SFB medium in a 96-well plate. Images were photographed using the AxionVision Rel.4.8 software of a Vert.A1 microscope (Zeiss, Oberkochen, Germany) in a 10x magnifying glass and analyzed using the Angiogenesis Analyzer plugin for ImageJ software v.1.52a.

### 4.8. Analysis of Cell Morphology

MDA-MB-231 cells (1 × 10^4^ cells/well) and HUVECs (3 × 10^4^ cells/well) were plated in a 96-well black microplate (Corning 3603) overnight at 37 °C, 5% CO_2_. Cells were exposed to Dis*Ba*-01 for 4 h in DMEM supplemented with 10% FBS in normoxia and hypoxia. Afterwards, cells were fixed in 3.7% paraformaldehyde for 10 min, permeabilized using 0.3% Triton X-100 for 5 min, and stained with Alexa Fluor^®^ 488 Phalloidin (F-actin dye, Thermo Fisher Scientific, Catalog Number: 12379) in DAPI-PBS (1:40) for 30 min. Fluorescent samples were observed using ImageXpress (Molecular Devices) equipment with 40x magnification. Morphology was analyzed in ImageJ software v.1.52a [65] and quantified using Threshold for MDA-MB-231 and ImageXpressMicro microscope (Molecular Devices, San Jose, CA, USA) under 40× magnification with the Meta-X-press software, and quantified using the Multi Wavelength Cell Scoringfor HUVEC.

### 4.9. Profile of β3 Integrin Subunit in Normoxia and Hypoxia by Flow Cytometry

Cells were incubated without and with Dis*Ba*-01 for 24 h in normoxia and hypoxia. Then, cells are harvested and centrifuged at 400 g in 4 °C. MDA-MB-231 cells and HUVECs were incubated with monoclonal integrin beta 3 antibody (ab11992, Abcam, Cambridge, UK), and washed and incubated with Alexa Fluor 488-labeled secondary antibody (ab11008, ThermoFisher, Waltham, MA, USA), followed by analysis in a flow cytometer (BD AccuriTM C6, BD Biosciences, Franklin Lakes, NJ, USA).

### 4.10. Apoptosis Assay

The possible apoptotic activity of Dis*Ba*-01 on MDA-MB-231 cells and HUVECs under hypoxia was analyzed by flow cytometry with the PE-Annexin V Apoptosis Detection Kit (BD Biosciences, Catalog Number: 559763). Cells (1 × 10^5^) were seeded in 24-well plates with DMEM and incubated overnight. A cell-free area was created using a sterile 200 µL pipette tip following treatment with or without mitomycin-c for 4 h for MDA-MB-231 and 2 h for HUVECs at 37 °C and 5% CO_2_. Then, cells were treated with Dis*Ba*-01 in medium containing 10% FBS and incubated in a normoxic and hypoxic environment for 24 h. After this period, control cells were harvested, heated at 100 °C for 5 min, and chilled at 4 °C immediately. Cells were incubated with PE-Annexin V and 7-aminoactinomycin D (7ADD) for 15 min in the dark at 4 °C, followed by the addition of binding buffer. Cells treated with Dis*Ba*-01 (0, 100, and 1000 nM) were incubated with PE-Annexin V and 7ADD, harvested, centrifuged at 400 g, and suspended in binding buffer. Analyses were performed in a flow cytometer (BD AccuriTM C6, BD Biosciences, Franklin Lakes, NJ, USA).

### 4.11. Statistical Analysis

Data were obtained in at least triplicate in three independent series of experiments and analyses were performed using the statistical SigmaPlot7 program. For parametric data, we performed two-way ANOVA or one-way ANOVA and post hoc Tukey test, and non-parametric data were subjected to the Kruskall–Wallis one-way analysis of variance on ranks post hoc Dunn test. Values of *p* < 0.05 were considered statically significant. Graphics were generated in the GraphPad program showing mean ± SD for normal distribution of population and median ± SD for non-normal distribution of population.

## Data Availability

The authors declare that the data generated in the current study are available within the article or from the corresponding author upon reasonable request.

## Supplementary Materials

The following supporting information can be downloaded at: https://www.mdpi.com/article/10.3390/ijms23031745/s1.
