# Supplementary figures and images for: The Effects of αvβ3 Integrin Blockage in Breast Tumor and Endothelial Cells under Hypoxia In Vitro
Source: Int J Mol Sci. 2022 Feb 3;23(3):1745. doi: 10.3390/ijms23031745 (PMC8835904; doi:10.3390/ijms23031745)

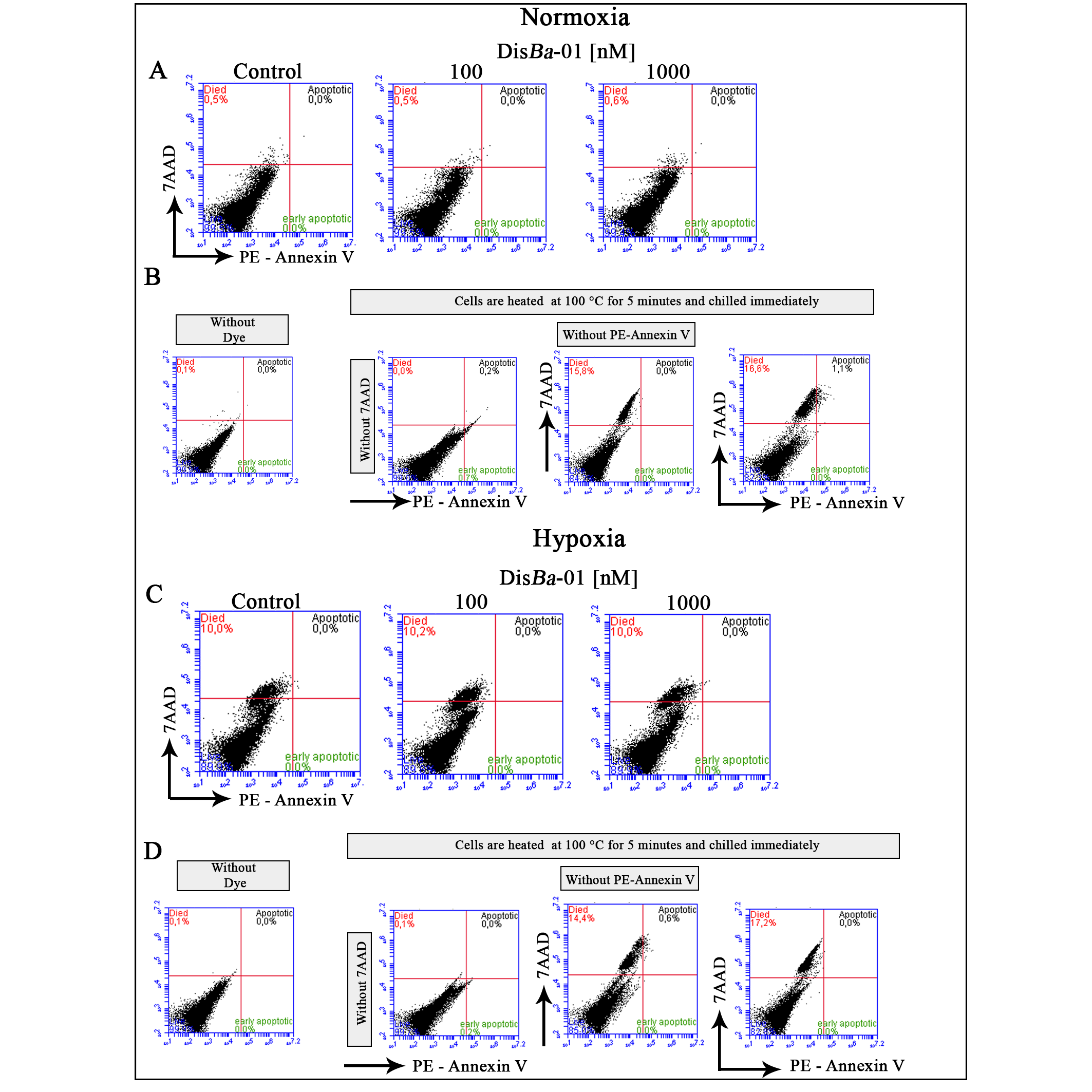

Supplement: Supplementary file 1 [file ijms-23-01745-s001.zip › Suppl Figure 1.tif]

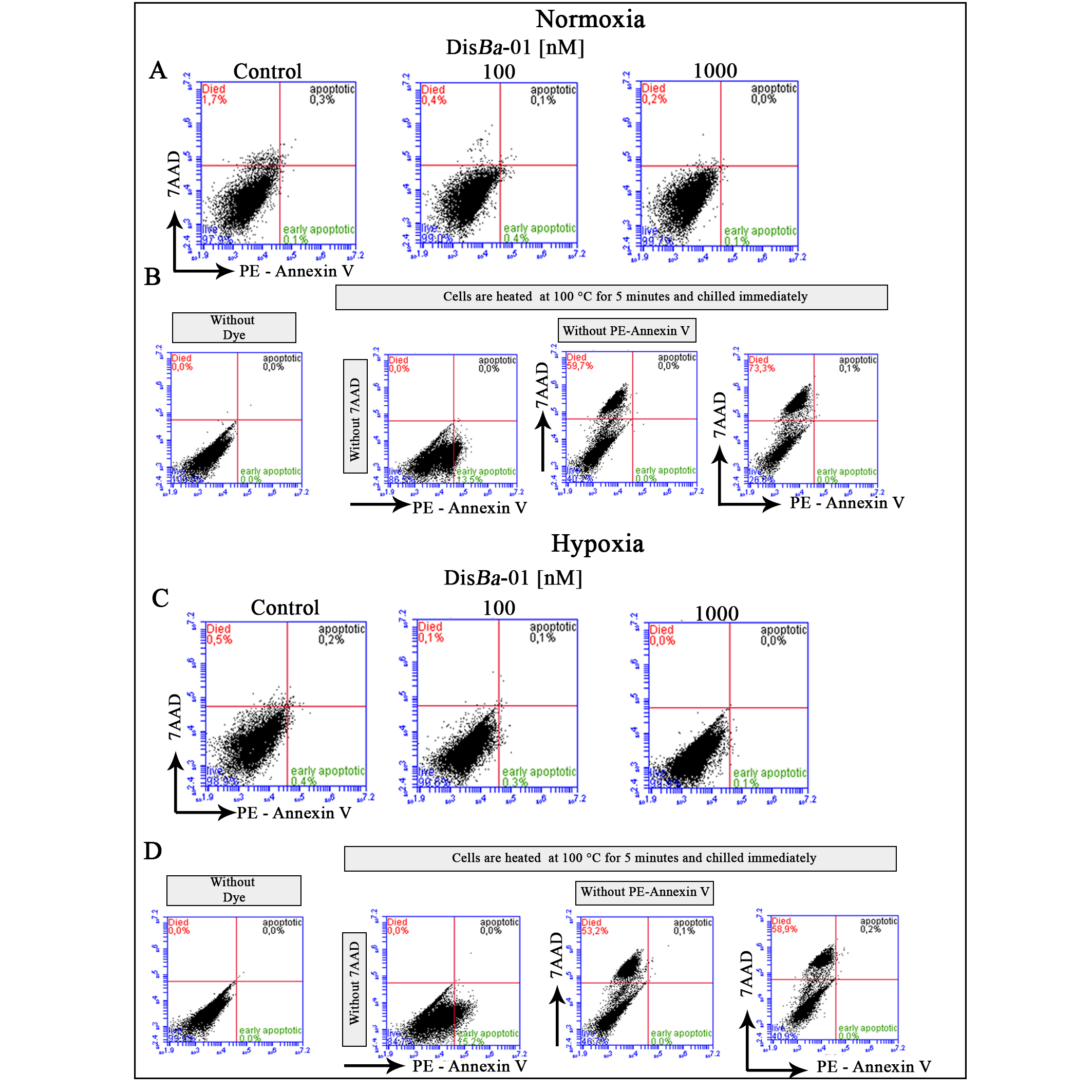

Supplement: Supplementary file 1 [file ijms-23-01745-s001.zip › Suppl Figure 2.tif]

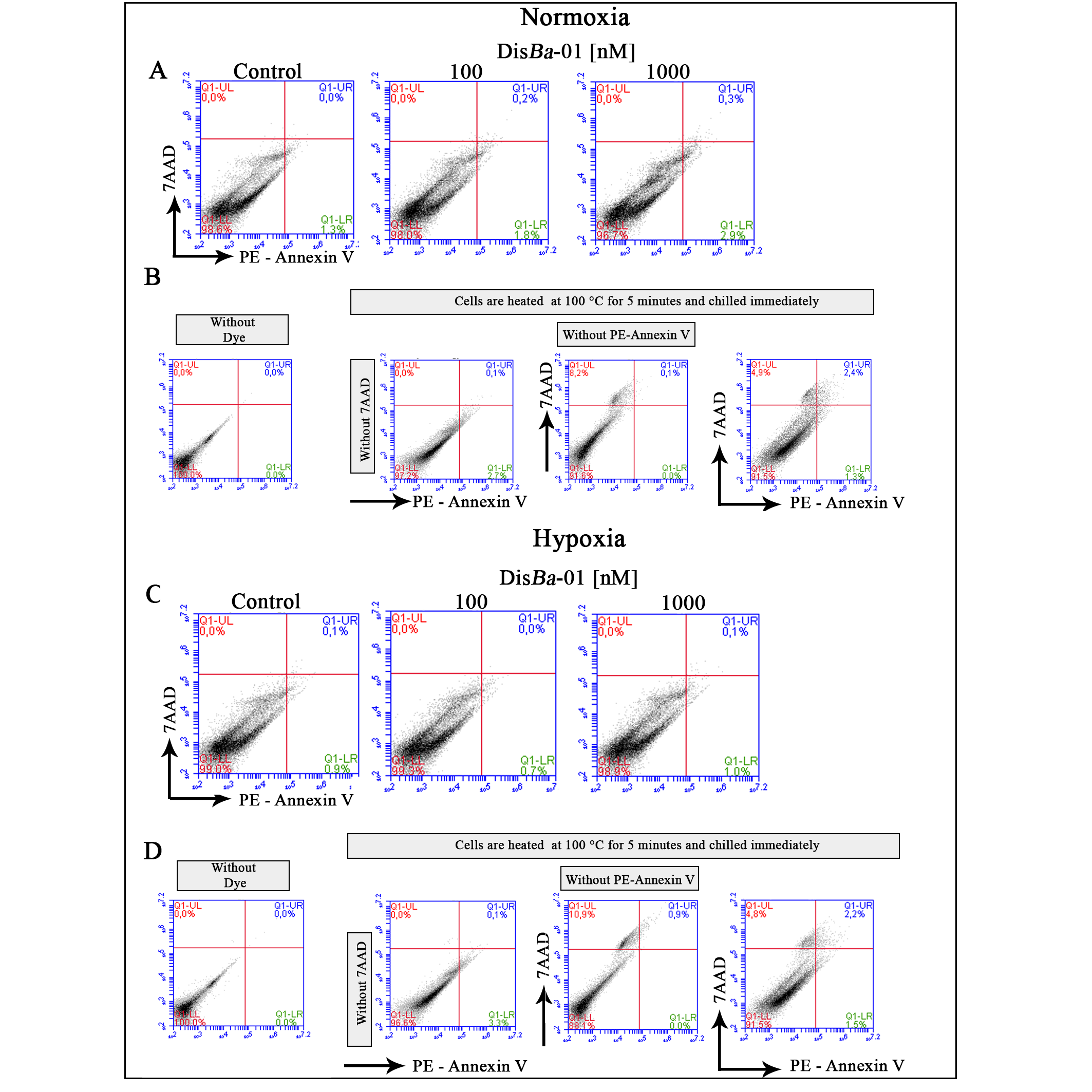

Supplement: Supplementary file 1 [file ijms-23-01745-s001.zip › Suppl Figure 3.tif]

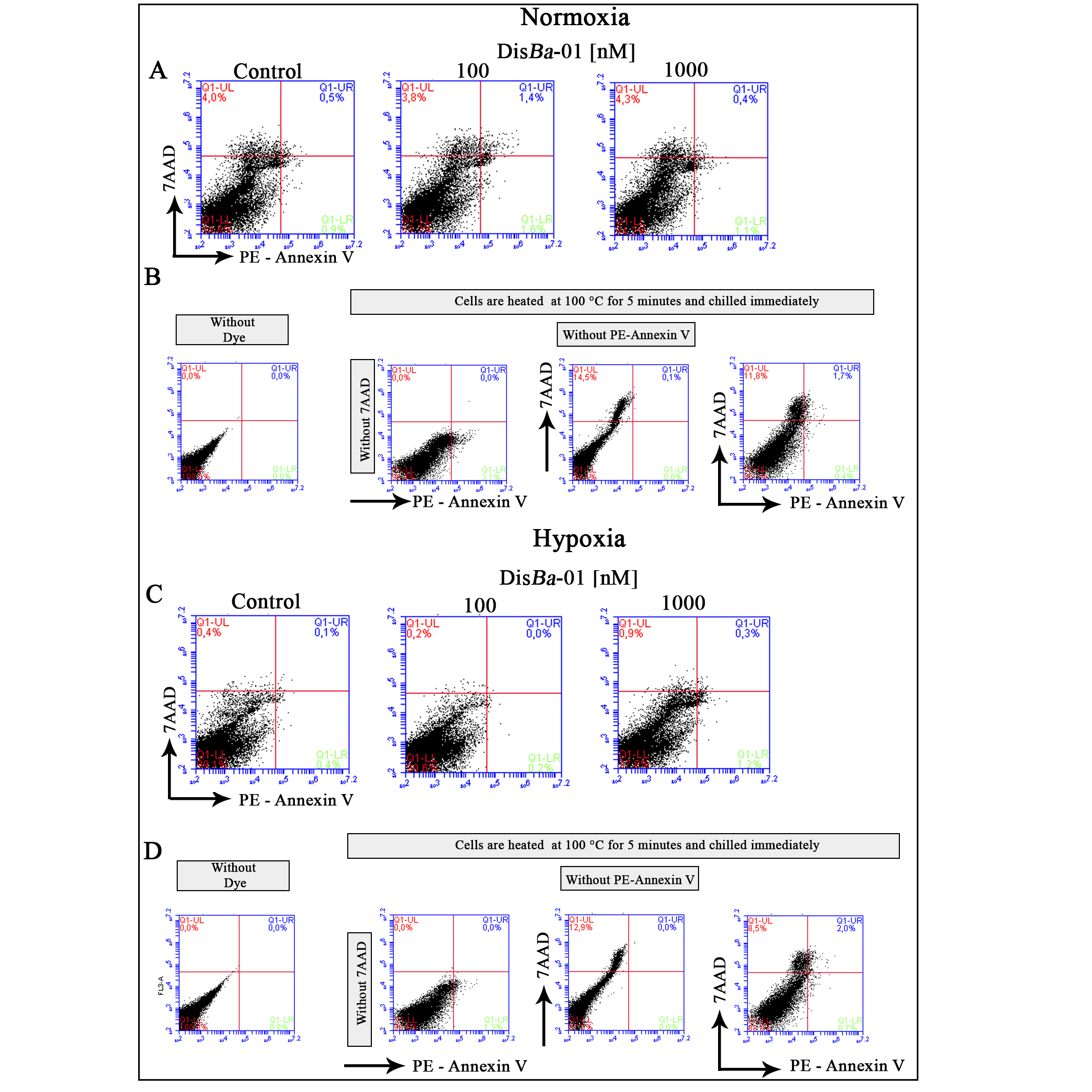

Supplement: Supplementary file 1 [file ijms-23-01745-s001.zip › Suppl Figure 4.tif]
